# Dr. Lawrence J. Gerold

**Published:** 1906-03

**Authors:** 


Dr. Lawrence J. Gerold, assistant surgeon of the State Soldier's Home Hospital at Bath, died March 15, 1906, from heart disease. He was forty-one years old, and was formerly assistant physician at the State Hospital, Kings Park, Long Island.

